# *Helicobacter pylori* infections in Ethiopia; prevalence and associated factors: a systematic review and meta-analysis

**DOI:** 10.1186/s12876-018-0927-3

**Published:** 2019-01-10

**Authors:** Addisu Melese, Chalachew Genet, Balew Zeleke, Tesfaye Andualem

**Affiliations:** 1 Department of Medical Laboratory Science, College of Medicine and Health Sciences, Bahir Dar University, Bahir Dar, Ethiopia; 2 Department of Nursing, College of Medicine and Health Sciences, Bahir Dar University, Bahir Dar, Ethiopia; 3 Department of Medical Laboratory Science, College of Health Sciences, Debre Tabor University, Debra Tabor, Ethiopia

**Keywords:** *Helicobacter pylori*, Systematic review, Meta-analysis, Ethiopia

## Abstract

**Background:**

*Helicobacter pylori (H.pylori)* infections are prevalent and recognized as major cause of gastrointestinal diseases in Ethiopia. However, Studies conducted on the prevalence, risk factors and other clinical forms of *H.pylori* on different population and geographical areas are reporting conflicting results. Therefore, this review was conducted to estimate the pooled prevalence of *H.pylori* infections and associated factors in Ethiopia.

**Methods:**

PubMed, Embase, Google scholar, and Ethiopian Universities’ repositories were searched following the Preferred Items for Systematic review and Meta-analysis (PRISMA) guideline. The quality of included studies was assessed using the Newcastle-Ottawa Scale in meta-analysis. Heterogeneity between studies was assessed using Cochrane Q test and I^2^ test statistics based on the random effects model. Comprehensive meta-analysis (CMA 2.0) and Review Manager (RevMan 5.3) were employed to compute the pooled prevalence and summary odds ratios of factors associated with of *H.pylori* infection.

**Results:**

Thirty seven studies with a total of 18,890 participants were eligible and included in the analysis. The overall pooled prevalence of *H.pylori* infection was 52.2% (95% CI: 45.8–58.6). In the subgroup analysis by region, the highest prevalence was found in Somalia (71%; 95% CI: 32.5–92.6) and the lowest prevalence was reported in Oromia (39.9%; 95% CI: 17.3–67.7). Absence of hand washing after toilet (OR = 1.8, 95% CI; 1.19–2.72), alcohol consumption (OR = 1.34, 95% CI; 1.03–1.74) and gastrointestinal (GI) symptoms (OR = 2.23, 95% CI; 1.59–3.14) were associated with *H.pylori* infection. The trend of *H.pylori* infection showed a decreasing pattern overtime from 1990 to 2017 in the meta-regression analysis.

**Conclusion:**

The prevalence of *H.pylori* infection remains high; more than half of Ethiopians were infected. Although the trend of infection showed a decreasing pattern; appropriate use of eradication therapy, health education primarily to improve knowledge and awareness on the transmission dynamics of the bacteria, behavioral changes, adequate sanitation, population screening and diagnosis using multiple tests are required to reduce *H.pylori* infections. Recognizing the bacteria as a priority issue and designing gastric cancer screening policies are also recommended.

**Electronic supplementary material:**

The online version of this article (10.1186/s12876-018-0927-3) contains supplementary material, which is available to authorized users.

## Background

*Helicobacter pylori* have been found to infect about half of the world’s population [1–5]. The prevalence of *H.pylori* infection varies globally with a greater prevalence generally reported from developing countries. The global estimate of *H.pylori* infection was reported at 48.5% while continental reports were 69.4% in South America, 37.1% in North America, 24.4% in Oceania, 54.6% in Asia, 47.0% in Europe and 79.1% in Africa [2, 6]. This difference has been related to geography, age, ethnicity, socioeconomic factors, and methods of diagnosis and eradication therapy [1, 6, 7]. Diseases associated with *H.pylori* infections are commonly occur at earlier ages in developing countries [1–3, 8–10].

The burden of *H.pylori* infections goes beyond the gastrointestinal tract and associated with different complications including hyperemesis gravidarum [11], coronary heart disease [12, 13], anemia [14–17], diabetes mellitus [18–22], cholecystitis [23, 24], HIV [25–27], growth trajectories [28], autoimmune and Parkinson’s disease [29]. Failure to *H.pylori* eradication therapy is also linked to bacterial resistance and poor patient compliance [30–34].

In 2017, World Health Organization (WHO) has published lists of 16 bacteria that pose the greatest risk for human health. *H.pylori* was thus categorized as a high priority pathogen for research and development of new and effective treatments [35]. In addition, recommendations are emerging to change approaches to management of *H.pylori* due to increased drug resistance [30, 31, 36–38]. The success of these developments needs knowledge of prevalence of *H.pylori*.

In Ethiopia, the prevalence of *H.pylori* infection ranged from 7.7% [39] to 91% [40]. It is highly prevalent and recognized as major cause of gastrointestinal diseases. Studies conducted on the prevalence, risk factors and other clinical forms of *H.pylori* on different population and geographical areas are reporting conflicting results. Socioeconomic factors, sanitation, crowded living conditions, unsafe food and water, ethnicity as well as poverty can contribute to *H.pylori* infections [41]. Studies published on the prevalence of *H.pylori* in Ethiopia dated back to the 1990’s [42]. However, comprehensive review has not been done on its prevalence and associated factors in Ethiopia. Therefore; this study was done to estimate the pooled prevalence of *H.pylori* infection and associated factors in Ethiopia.

## Methods

### Data bases and search strategy

**Fig. 1:**
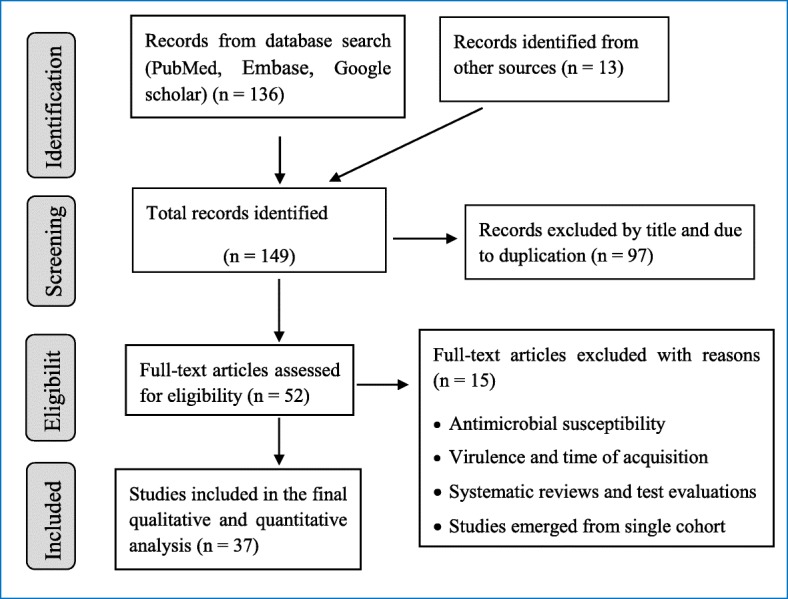
PRISMA flow chart of article selection

PubMed, Embase, and Google scholar were searched to identify potential articles on *H.pylori* infections in Ethiopia. To include unpublished studies, Ethiopian University repositories were searched and reference lists of eligible studies were searched to maximize inclusion of relevant studies. The search was conducted following the PRISMA guideline and checklists ( [43], Fig. 1). The following terms with MeSH (Medical Subject Headings) and Boolean operators were used to search PubMed; *Helicobacter pylori* OR *H.pylori* OR *Campylobacter pylori* OR *C.pylori* OR gastritis OR gastric cancer OR gastric carcinoma OR peptic ulcer disease OR PUD OR duodenal ulcer OR dyspepsia OR mucosa associated lymphoid tissue OR MALT AND Ethiopia. The search was limited to English language publications and done independently by each reviewer to minimize bias and the missing of studies. Search results were combined in to EndNote X6 file (Clarivate Analytics USA) and duplicates were removed. All articles published up to June 30, 2018 were included in the review if fulfilled the eligibility criteria (Table 1).

**Table 1:** Eligibility criteria

Inclusion criteria	Exclusion criteria
• Country and setting: Ethiopia and any setting • Study design: any study design • Outcome: reported the prevalence or number of *H.pylori* cases and sample size • Type of articles: full text and peer-reviewed; published in English language • Publication year: up to June 30, 2018 • Types of diagnosis: reported any laboratory tests	• Evaluation of diagnostic tests and antimicrobial sensitivity • Systematic reviews and meta-analysis • Studies on virulence factor genes • Studies on age at acquisition of *H.pylori* infection and its effect • Studies emerged from single cohort study with similar prevalence report

### Quality assessment

The quality of included studies was assessed by using the Newcastle-Ottawa Scale (NOS) (Additional file 1). Two authors independently assessed the quality of each study. Disagreements between authors was resolved by discussion and articles were included if agreement was reached between the authors.

### Data extraction

Data were extracted into customized Microsoft Excel. Data extracted from each included study include; author, publication year, study area, study period, study design, study population, sample size, laboratory tests used, prevalence and/or number of *H.pylori* cases. We have also contacted corresponding authors of the included studies for missing data though no one responded.

### Data analysis

Studies providing data on crude prevalence of *H.pylori* or numbers of cases and study participants were included in the meta-analysis. Prevalence for individual studies was determined by multiplying the ratio of cases to sample size by 100. The estimation of pooled prevalence and summary odds ratios *of H.pylori* infection was done using CMA 2.0 and RevMan 5.3 softwares. Subgroup analyses were done by study period, study region, study design, laboratory tests used (types and numbers) and publication history. With the assumption that true effect sizes exists between eligible studies, the random effects model was used to determine the pooled prevalence, summary odds ratios and 95% CIs. Significant association between *H.pylori* infections and potential factors was declared at *p*-value < 0.05. Heterogeneity was evaluated using the Cochran’s Q test and I^2^ statistics. Significant heterogeneity was declared at I^2^ > 50% and Q-test (*P* < 0.10).

### Publication bias and sensitivity analysis

Funnel plots were drawn to assess the possibility of publication bias. We plotted the studies’ logit event rate and the standard error to detect asymmetry in the distribution. A gap in the funnel plot indicates potential for publication bias. In addition, Begg’s adjusted rank correlation and Egger’s regression asymmetry tests were used to assess publication bias, with *P* < 0.05 considered to indicate potential publication bias. Sensitivity analysis, leave-one-out analysis was done to assess the prime determinant of the pooled prevalence of *H.pylori* infection and to detect the possible causes of heterogeneity between studies.

## Results

### Characteristics of included studies

Thirty seven studies with a total population of 18,890 met the inclusion criteria and included in the analysis. The detail characteristics of included studies are shown in (Table 2). The included studies were conducted between 1990 and 2017; of which 29 were published and 8 were unpublished. Thirteen studies were conducted in the Southern Nations, Nationalities and Peoples Region (SNNPR) of Ethiopia. Eleven studies were conducted in Addis Ababa; nine studies in Amhara region, two studies in Oromia, one study in Somalia and one study in Benishangul Gumuz region. Nineteen studies were reported among adults [15, 39, 40, 42, 44–58], eight of the studies were reported on children [14, 59–65] while nine studies were conducted on both adults and children [25, 26, 58, 66–72]. One study did not describe the study population [73]. Among the include studies, 20 were cross-sectional, eight were case control, six were cohort, and two were retrospective studies. Ten studies used multiple tests in detecting *H.pylori* while 27 studies employed a single test to declare *H.pylori* infection. The prevalence of *H.pylori* from eligible individual studies ranged from 7.7 to 91.0%.

**Table 2:** Lists and characteristics of the included 37 studies

Author, year	Study period	Study region	Study design	Study subjects	Lab test used	Sample size	Cases	Prevalence (%)	Publication history
Ababu, 2016	2016	Addis Ababa	Cross-sectional	HIV patients on ART	Stool antigen	388	213	54.9	Unpublished
Abebaw, 2014	2013	Amhara	Cross-sectional	Dyspeptic patients	Serology (IgG)	209	151	72.2	Published
Alebie, 2016	2016	Somalia	Cross-sectional	University students with gastritis	Serology (IgG, IgM, IgA)	145	103	71.0	Published
Alemayehu, 2011	2010–2011	South	Case control	Dyspeptic and non-dyspeptic patients	Serology (IgG)	106	66	62.3	Unpublished
Amberebir, 2011	2008–2009	South	Cohort	Children at age three	Stool antigen	616	253	41.1	Published
Amberebir, 2014	2008–2009	South	Cohort	Children at age five	Stool antigen	857	377	44.0	Published
Asrat, 2004	2000–2002	Addis Ababa	Cross-sectional	Dyspeptic patients	PCR, culture, Rapid Urease,, Histology, Silver stain, stool antigen, serology (EIA, immunoblot – IgG),	300	273	91.0	Published
Assefa, 2017	2016	Addis Ababa	Case control	Pregnant women	Stool antigen	150	37	24.7	Unpublished
Ayele, 2017	2016	South	Case control	Dyspeptic and non-dyspeptic patients	Stool antigen	168	13	7.7	Published
Berhaneselassie, 2017	2010	South	Case control	Dyspeptic and non-dyspeptic patients	Serology (IgG)	195	175	89.7	Published
Desta, 2002	2001	Addis Ababa	Cross-sectional	Blood donors	Serology (IgG)	150	133	88.7	Published
Dilnessa, 2017	2015	Gumuz	Cross-sectional	Dyspeptic and non-dyspeptic patients	Stool antigen	230	112	48.7	Published
Hailu. 2016	2012–2013	South	Cross-sectional	Upper GI symptoms	Stool antigen	349	177	50.7	Published
Henriksen, 1999	1992–1995	South	Cohort	PUD and non-PUD patients	Rapid urease, Loffler stain	290	234	80.7	Published
Kassew, 2017	2016	Amhara	Cross-sectional	Dyspeptic patients	Stool antigen	354	133	37.6	Published
Kebede, 2015	2014	Oromia	Cross-sectional	TB and non-TB patients	Stool antigen	108	20	18.5	Published
Kemal, 2014	2014	Addis Ababa	Cross-sectional	Upper GI symptoms	Stool antigen	221	57	25.8	Unpublished
Kibru, 2014	2013	South	Cross-sectional	Dyspeptic patients	Stool antigen	401	210	52.4	Published
Lindkvist, 1998	1995	South	Case control	Rural and 2–4 years old children	Serology(EIA, immunoblot - IgG)	242	116	47.9	Published
Lindkvist, 1999	1995	South	Cohort	Seronegative children	Serology(EIA, immunoblot - IgG)	77	44	57.1	Published
Mathewos, 2013	2009–2011	Amhara	Retrospective	H.pylori suspects	Serology (IgG, IgM, IgA)	1388	912	65.7	Published
Moges, 2006	2003	Amhara	Cross-sectional	Dyspeptic patients	Serology (IgG)	215	184	85.7	Published
Seid, 2017	2017	Addis Ababa	Case control	Dyspeptic and non-dyspeptic HIV patients	Stool antigen	370	117	31.6	Unpublished
Seid, 2018	2015	Amhara	Cross-sectional	Upper GI symptoms	Stool antigen	318	99	31.1	Published
Seid, 2018a	2015–2016	Amhara	Cross-sectional	Upper GI symptoms	Serology (IgG)	363	255	70.2	Published
Seid, 2018b	2016	Amhara	Cross-sectional	Upper GI symptoms	Stool antigen, serology (IgG)	342	104	30.4	Published
Tadege, 2005	2002–2003	Amhara	Case control	Dyspeptic and non-dyspeptic patients	Serology (EIA, imunoblot - IgG)	200	124	62.0	Published
Tadesse, 2011	2009	Addis Ababa	Case control	Dyspeptic and non-dyspeptic patients	Stool antigen, serology (IgG, IgM, IgA)	238	109	45.8	Published
Tadesse, 2014	2012–2013	South	Cross-sectional	Upper GI symptoms	Serology (double ELISA -IgG)	408	340	83.3	Published
Taye, 2015	2008–2009	South	Cohort	Children at age 6.5	Stool antigen	848	88	10.4	Published
Tedla, 1992	1990	South	Cohort	Upper GI symptoms	Rapid urease, Loeffler, Methylene blue stain	444	324	73.0	Published
Teka, 2016	2010–2011	Addis Ababa	Cross-sectional	HIV positive and negative patients	Serology (IgG)	212	120	56.6	Published
Terfa, 2015	2015	Addis Ababa	Cross-sectional	Women of child bearing age	Stool antigen	332	96	28.9	Unpublished
Tesfaye, 2017	2016–2017	Oromia	Cross-sectional	Health facility and school children	Serology (IgM, IgG, IgA), stool antigen	461	296	64.2	Unpublished
Tsega, 1996	1994	Addis Ababa	Case control	NUD and asymptomatic patients	Gram, giemsa, gemineze stain	207	120	58.0	Published
Workineh, 2016	2009–2013	Amhara	Retrospective	Dyspeptic patients	Serology (IgG, IgM, IgA)	6566	2733	41.6	Published
Worku, 2017	2017	Addis Ababa	Cross-sectional	School children	Stool antigen	422	61	14.5	Unpublished

### Pooled prevalence of *H.pylori*

**Fig. 2:**
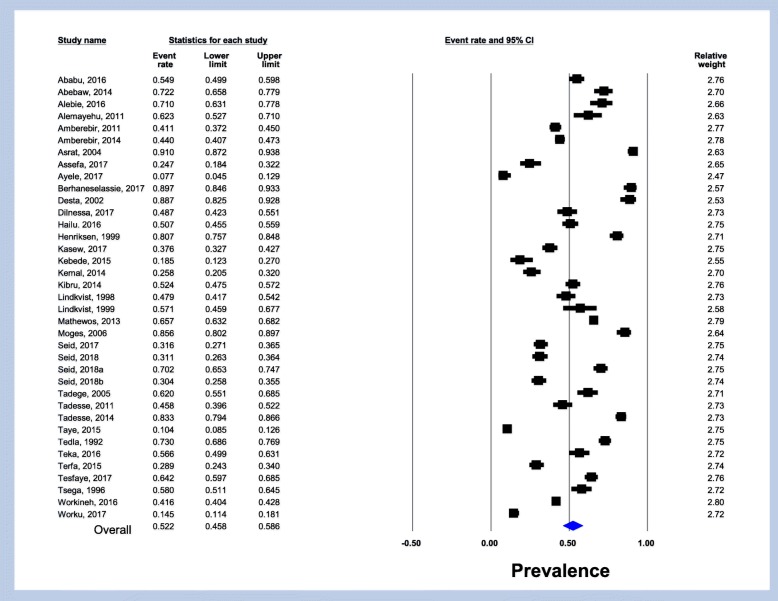
Forest plot of the pooled prevalence of *helicobacter pylori* infection in Ethiopia from 37 studies

**Fig. 3:**
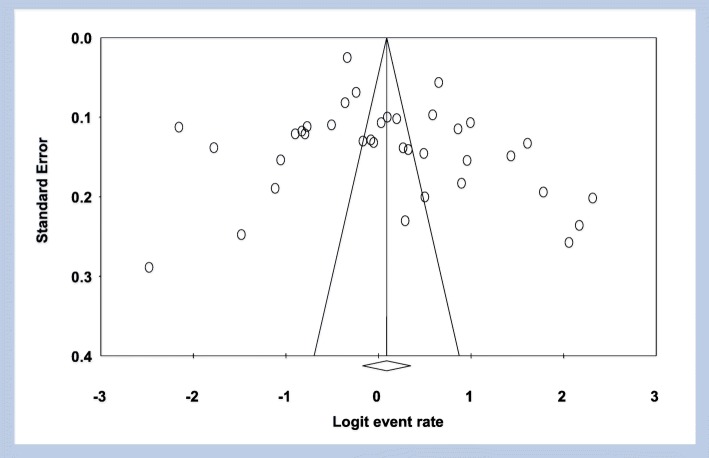
Publication bias assessment funnel plot; Egger’s regression test (*p* = 0.172) and Begg’s rank correlation (*p* = 0.367)

A total of 18,890 Ethiopians were participated in the study; out of which 8979 were infected with *H.pylori* in the period under review giving an overall pooled prevalence of 52.2% (95% CI: 45.8–58.6; I^2^ = 51.05%, *p* = 0.503) (Fig. 2). Sensitivity analysis revealed no significant difference both in the pooled prevalence and heterogeneity. When one study was excluded from the analysis step-by-step, the pooled prevalence was between 50.5 and 53.5% while heterogeneity was similar (I^2^ = 53.5%). Drawing of funnel plot supported with Egger’s regression (*p* = 0.172) and Begg’s correlation (*p* = 0.367) tests showed no evidence of significant publication bias (Fig. 3).

### Subgroup prevalence of *H.pylori*

Prevalence of *H.pylori* for subgroups was analyzed for study region, study period, sample size, study design, type and number of diagnostic tests used and publication history. The prevalence of *H.pylori* when studies were categorized by region ranged from 39.9% (95% CI: 17.3–67.7%; I^2^ = 67.6%, *P* = 0.486) in Oromia to 71% (95% CI: 32.5–92.6.2; I^2^ = 0.0%, *P* = 0.280) in Somalia. Other regional prevalence rates were 48.1% in Addis Ababa, 54.6% in Amhara, 48.7% in Benishangul Gumuz and 53.6% in SNNPR. Subgroup analysis by publication history showed a prevalence of 56.5% from published and 36.8% from unpublished studies. Subgroup analysis was also computed by the study period when the studies were conducted to see the trend of *H.pylori* infection. Hence, the prevalence of *H.pylori* was 64.4% in the period 1990–2000, 62.2% in the period 2001–2011 and 42.9% in the period 2012–2017, showing a decreasing trend. The pooled prevalence was also higher in studies which used multiple tests than studies employed a single test to detect *H.pylori* infection (62.9 and 48.1%, respectively) (Table 3).

**Table 3:** Pooled prevalence of *H.pylori* infections in Ethiopia stratified according to sub-groups

Variables	No of included studies	Pooled prevalence estimate			Heterogeneity	
		Sample size	Cases	Prevalence, 95% CI	I^2^ (%)	Q (P-value)
Region						
Addis Ababa	11	2990	1336	48.1 (36.3–60.2)	50.4	0.790
Amhara	9	9955	4695	54.6 (42.0–66.7)	1.7	0.412
Benishangul gumuz	1	230	112	48.7 (16.0–82.6)	0.0	0.954
Oromia	2	569	316	39.9 (17.3–67.7)	61.4	0.517
Somalia	1	145	103	71.0 (32.5–92.6)	–	–
SNNPR	13	5001	2417	53.6 (42.4–64.4)	53.1	0.569
Study period ^a^						
1990–2000	5	1260	838	64.4 (47.3–78.5)	0.0	0.097
2001–2011	12	8724	4118	62.2 (51.1–72.1)	64.3	0.031
2012–2017	21	8906	4.023	42.9 (34.8–51.4)	37.0	0.102
Sample size						
< 150	6	736	403	54.7 (36.8–71.4)	51.3	0.614
151–500	26	7879	4213	54.5 (45.9–62.8	39.5	0.306
501–1000	3	2321	718	28.6 (12.8–52.2)	31.2	0.074
> 1000	2	7954	3645	53.9 (25.5–79.9)	0.0	0.804
Study design						
Cross-sectional	20	5928	3137	55.1 (44.9–64.9)	39.9	0.324
Case control	9	1876	877	47.0 (32.5–62.1)	44.7	0.704
Cohort	6	3132	1320	49.7 (39.6–67.5)	45.7	0.972
Retrospective	2	7954	3645	53.9 (24.5–80.5)	0.0	0.812
Lab tests used						
Stool antigen	18	6712	2276	31.4 (24.6–39.1)	8.5	< 0.001
Serology (IgM, IgG, IgA)	15	10,937	5752	69.7 (61.2–76.9)	2.3	< 0.001
Others ^b^	4	1241	951	77.8 (63.0–87.8)	21	0.001
Number of tests used						
Single	27	7235	16,099	48.1 (40.8–55.5)	57.22	0.662
Multiple	10	1744	2801	62.9 (51.0–73.4)	19.7	0.034
Publication history						
Published	29	16,440	8036	56.6 (49.2–63.6)	51.5	0.081
Unpublished	8	2450	943	36.8 (24.9–50.5)	11.2	0.059

*SNNPR* South nations, nationalities and peoples region

^a^ One study is divided in to two datasets by study period making the total dataset 38

^b^ Includes (PCR, culture, rapid urease, methylyne blue stain, giemsa stain, loefler stain, histopatholgy, silver stain, gemnieze stain, gram stain,….)

### Factors associated with *H.pylori* infection

Factors associated with *H*.*pylori* infections were grouped in to; sociodemographic, environmental, behavioral and clinical factors. Summary odds ratios (ORs) and their respective 95% confidence intervals (CIs) were computed based on the random effects model. Whenever there was data from the studies, we have tried to summarize the ORs and 95% CIs to identify the factors associated with *H.pylori* infections.

#### Socio-demographic risk factors

**Fig. 4:**
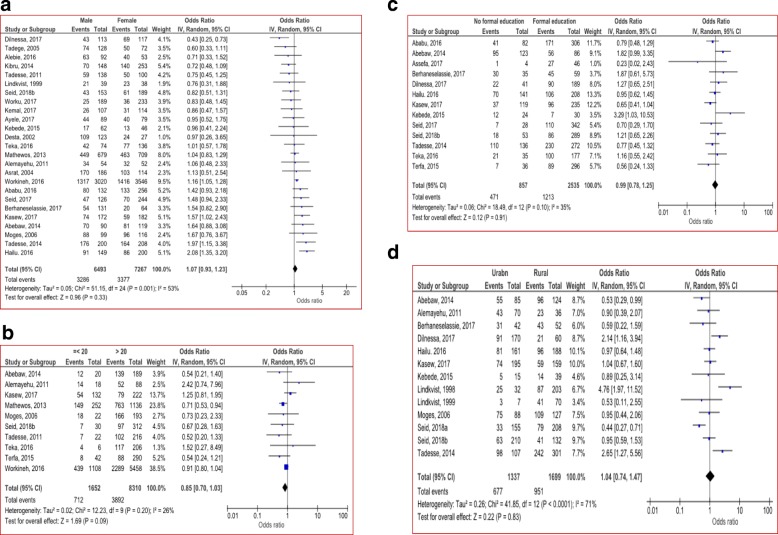
Sociodemographic factors associated with *H.pylori* infection by sex (**a**); by age group (**b**); by educational level (**c**) and by residency (**d**)

Available sociodemographic data (by age, sex, residency and level of education) were extracted and analyzed to determine their possible association with *H.pylori* infection but none of these variables had significant association. Even though not significant; male participants (OR = 1.07; 95% CI: 0.93–1.23; *p* = 0.33) and urban residents (OR = 1.04; 95% CI: 0.74–1.74; *p* = 0.83) were more likely to be infected with *H.pylori* than their counter parts (Fig. 4).

#### Environmental factors

**Fig. 5:**
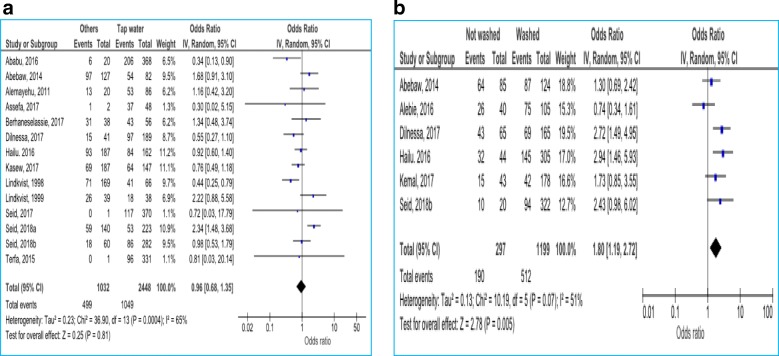
Environmental factors associated with *H.pylori* infection by type of water source for drinking (**a**); and by hand washing habit after toilet (**b**)

Sources of drinking water, hand washing before meal and after toilet were the environmental factors assessed for their possible association with *H.pylori* infection. Participants who were not washing their hands after toilet were more likely to be infected with *H.pylori* (OR = 1.8; 95% CI: 1.19–2.72; *p* = 0.005). Other variables had no significant association (Fig. 5).

#### Behavioral factors

**Fig. 6:**
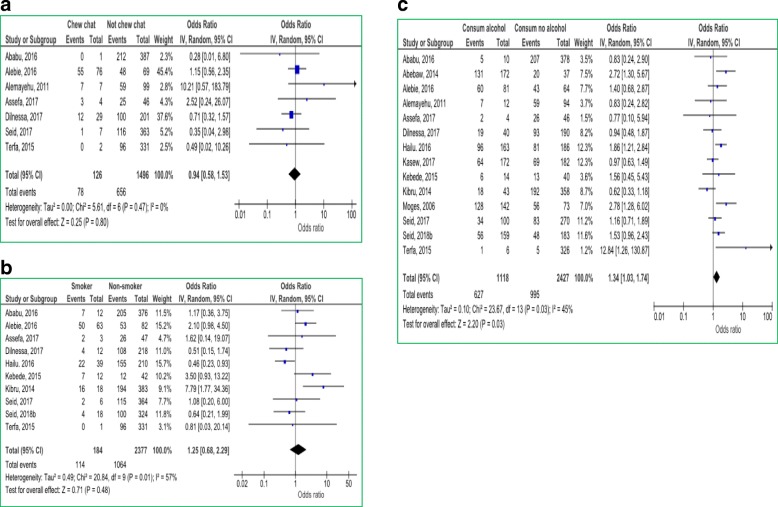
Behavioral factors associated with *H.pylori* infection chat chewing (**a**); by cigarette smoking (**b**) and by alcohol consumption (**c**)

Chat chewing, cigarette smoking and drinking alcohol were analyzed for any association with *H.pylori* infection. Even though not significant, chat chewing had a preventive effect (OR = 0.94; 95% CI: 0.58–1.53; *p* = 0.80) for *H.pylori* infection while smoking increases the risks of infection (OR = 1.25; 95% CI: 0.67–2.30; *p* = 0.48). Participants who were taking alcohol had a significant association with *H*.*pylori* infection (OR = 1.34; 95% CI: 1.03–1.74; *p* = 0.03) (Fig. 6).

#### Clinical factors

**Fig. 7:**
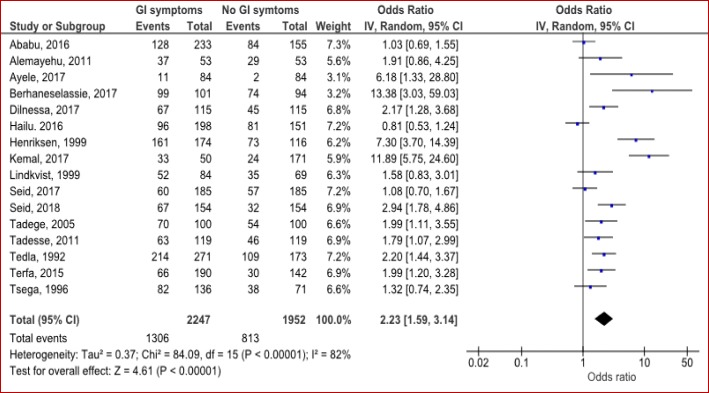
Clinical factors associated with *H.pylori* infection by gastrointestinal (GI) symptoms

Gastrointestinal (GI) symptoms, allergic reactions, hyperemesis gravidarum, HIV and TB infections were some of the clinical factors reported with *H.pylori* infection by included studies. Because studies on allergic reactions, hyperemesis gravidarum, HIV and TB infections are small enough to compute pooled summary of odds ratios, we analyzed only the association between GI symptoms and *H.pylori* infection. Hence; participants who had GI symptoms (including dyspepsia, gastritis, peptic ulcer and related) were more likely to be infected with *H.pylori* (OR = 2.23; 95% CI: 1.59–3.14; *p* < 0.00001) (Fig. 7).

## Meta-regression

**Fig. 8:**
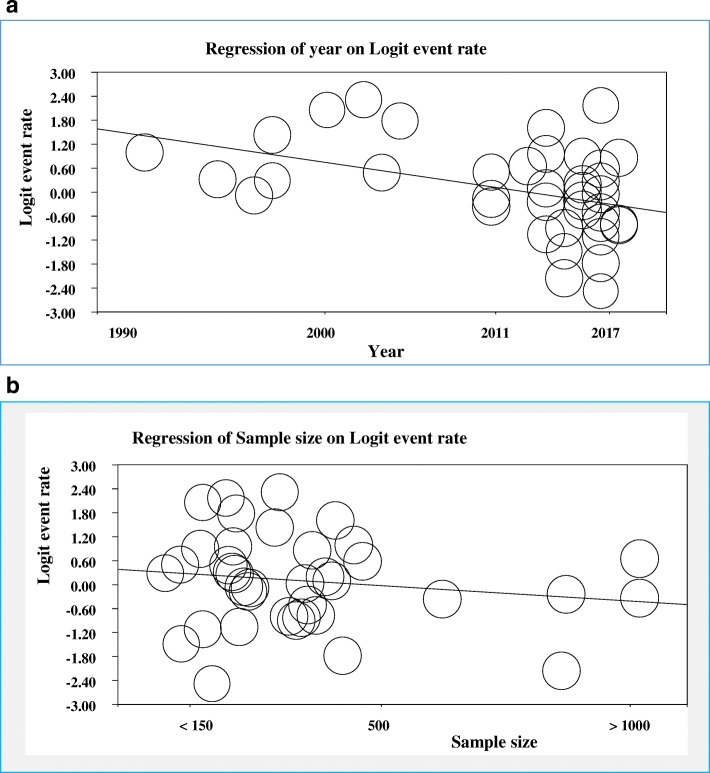
Meta-regression of the prevalence *H.pylori* infection by year of study (B = −0.067, *p* = 0.00004) (**a**); and sample size of studies (B = −0.00079, *p* = 0.193) (**b**)

Meta-regression was done to explore the trend of prevalence of *H.pylori* by year of study and sample size of the included studies. A significant downward trend of *H.pylori* infection was observed from 1990 to 2017 (B = − 0.067, *p* = 0.00004). However; there was no significant association between prevalence of *H.pylori* and sample size of the studies even though there was a slight decrease of prevalence of H.pylori with increased sample size (B = − 0.00079; *p* = 0.193) (Fig. 8).

## Discussion

The global prevalence of *H.pylori* infection was estimated at 48.5% in 2017 [6]. The World Gastroenterology Organization (WGO) in its 2011 global guideline reported prevalence of > 95% among adults, 48% among 2–4 age groups and 80% among children aged at six in Ethiopia [1] but there was no national pooled prevalence reported yet. Estimating the national and regional prevalence, trends of infection and associated factors is crucial to establish appropriate strategies for the diagnosis, prevention and control of *H.pylori* infection. Estimates of *H.pylori* infection is usually challenging since some factors have profound effect than others and some studies look in to distinct population or samples, method of isolation, geographical distribution, socioeconomic, behavioral, environmental and clinical factors.

The 37 studies included in our analysis determined the prevalence of antigens and/or antibodies (either IgM, IgA or IgG) of *H.pylori* and ranged from 7.7 to 91.0% among different study populations, geographical areas and study period. However, the overall pooled prevalence of *H.pylori* in Ethiopia was estimated to be 52.2% (95% CI: 45.8–58.6). This overall prevalence estimate is lower than reports from Nigeria (87.7%), South Africa (77.6%), Portugal (86.4%), Tunisia (72.8%), Brazil (71.2%) and Estonia (82.5%) [6]. Some reasons may explain the lower prevalence in Ethiopia. Firstly; the number of studies and participants included; pooled from 37 studies and 18,890 participants in our analysis compared to fewer number of studies and participants. Second, our analysis included recent reports which showed a decreasing trend to recent times. Third, most of the laboratory tests used by studies included in our review were based on stool antigen for detection of *H.pylori*; which has low isolation rate.

However, our estimate is higher than other countries; 22.1% in Denmark, 43.6% in Thailand, 46.8% in Democratic republic of Congo (DRC) and 40.9% in Egypt [6]. These differences might be attributable to differences in time trend of studies, poor personal and environmental hygiene, low socioeconomic status and behavioral factors; and sensitivity/specificity of laboratory tests employed in detecting *H.pylori*. Stool antigen and serological tests were the most widely used methods used in detecting *H.pylori* infection*.*

The trend of *H.pylori* infection showed a decreasing pattern in the last three decades from 1990 to 2017; 64.4% in the first decade (1990–2000), 62.2% in the second decade (2001–2011) and 42.9% in the third decade (2012–2017). This decrement might be related with relative improvements in sanitation, water access, life style and behavioral changes, quality of life and socioeconomic status, and increased awareness on the transmission, diagnosis, eradication therapy, prevention and control of *H.pylori* infection.

Regional estimates of *H.pylori* infection in the subgroup analysis showed a lower prevalence of 39.9% in Oromia and higher prevalence of 71.0% in Somalia region. This regional difference can be attributable that in Somalia, study participants were University students with known gastritis which is a known risk factor for *H.pylori* infection. Other explanations could be related with sociodemographic, socioeconomic, environmental, clinical, behavioral factors and number of studies included in each category.

The prevalence of *H.pylori* infection differs on the bases of laboratory tests used. Higher prevalence was observed when detection is supplemented with sensitive tests including PCR, culture, rapid urease test and histopathology as shown in (Table 3). This is supported by studies comparing diagnostic tests for *H.pylori* that sensitive tests improve the detection rate of *H.pylori* infections from clinical samples [74–77]. In addition, combination of at least two diagnostic methods is recommended to increase the validity of results [16, 20, 28, 48, 63, 78–82], but only ten studies used multiple tests to make a definitive diagnosis of *H.pylori* in our analysis. Our subgroup analysis confirms that the pooled prevalence of *H.pylori* infection when multiple tests are used is higher than the pooled prevalence when a single test is used to detect *H.pylori* infection (62.9% Vs 48.1%).

Several risk factors for *H.pylori* infection were identified and reported usually with conflicting result. These factors are to be analyzed and pooled to have a summary of effect sizes. The results of this meta-analysis showed that participants with gastrointestinal (GI) symptoms were more likely to be infected with *H.pylori* (OR = 2.23; 95% CI: 1.59–3.14). This could be due to the effect of GI symptoms providing a growing medium (changing pH, thinning of gastric wall, gastric ulceration, change in gut microbiota) for the bacteria.

Although the type and level of alcohol, amount and frequency of consumption were not described, individuals taking alcohol were more likely to be infected with *H.pylori* infection than those who did not consume alcohol. This result is inconsistent with previous studies [83–86] reporting that alcohol consumption has either a protective effect or has no any relation with *H.pylori* infection. Other study [87] reported alcohol as a risk factor for *H.pylori* supporting our analysis. As alcohol is known to directly damage the gastric mucosal layer, it is theoretically possible that alcohol can provide ways for *H.pylori* infection. In addition, heavy drinking can possibly predispose consumers to social contacts that favor transmission of *H.pylori* infection. Other mechanism may be involved in the synergistic effect of alcohol including bacterial adherence and host factors in facilitating infection among drinkers. Further longitudinal and epidemiological studies are needed to test these explanations.

Previous studies [1, 86, 88] have reported that the prevalence of *H.pylori* infection seems to increase with age but the increment with age is assumed most likely due to cohort effect. As most infections are acquired early in life; the bacteria usually persists indefinitely unless treated with specific antibiotic. In our analysis, consistent with the assumption, participants in the age of <20 years were less likely to be infected when compared to above 20 years of age but the association is not significant (OR = 0.85; 95% CI: 0.70–1.03).

Poor sanitation and unsafe food and water were repeatedly reported as risk factors contributing for *H.pylori* infection. In our analysis, individuals who were not washing their hands after toiled were more likely to be infected with *H.pylori*. This is in agreement with previous studies [1, 41] and can be explained that *H.pylori* is largely transmitted through feco-oral or oral-oral routes. Lack of proper sanitation and basic hygiene after toilet, therefore; can be source of infection and increase the chance of acquiring *H.pylori*. World Health Organization (WHO) has also identified it as one of the greatest risks for human health and categorized it as a high priority pathogen for research and development of new and effective treatments [35].

In the meta-regression analysis, the prevalence of *H.pylori* infection showed a significant downward trend from 1990 to 2017 (B = − 0.067, *p* = 0.00004). This can be due to relative improvements in sanitation, water access, lifestyle and behavioral changes, quality of life and socioeconomic status, increased awareness on the transmission, diagnosis, eradication therapy, preventive and control of *H.pylori* infection. On the other hand; a slight decrement of prevalence of *H.pylori* was observed with increased sample size (B = − 0.00079; *p* = 0.193) but the association was not significant. This might be related with the numbers of studies done on large sample sizes in that only few studies were done when compared to numbers of studies done on small sample sizes.

## Strengths and limitations

The strength of this study is that it included relatively larger numbers of published and unpublished studies without time limit of publication year. It has provided the national pooled prevalence, trends of infection and identified factors associated with *H.pylori* infection. The study included sub-group analysis and meta-regression of differences of study area, laboratory tests, study period, sample size and study designs. In few regions of Ethiopia, no study was found from and most identified studies were hospital based which could affect the generalisability of our findings. Moreover, there was lack of data sets to investigate the association between *H.pylori* infection and possible risk factors and the outcome variables may be affected by other cofounders.

## Conclusion

The prevalence of *H.pylori* infection remains high; more than half of Ethiopians were infected and significant association was observed between *H.pylori* infection and absence of hand washing after toilet, alcohol drinking and presence of gastrointestinal (GI) symptoms. These findings strengthen the action to implement the control and prevention of *H.pylori* infection more effectively to prevent gastric cancer and other related complications in Ethiopia. Although the trend of infection showed a decreasing pattern; appropriate use of eradication therapy, health education primarily to improve knowledge and awareness on the transmission dynamics of the bacteria, behavioral changes, adequate sanitation, population screening and diagnosis using multiple tests are required to reduce *H.pylori* infections.. Recognizing the bacteria as a priority issue and designing gastric cancer screening policies are also recommended.

## Additional file

Additional file 1: Newcastle-Ottawa Scale for cross sectional studies. (DOC 34 kb)

## Abbreviations

EIA: Enzyme immune assay
ELISA: Enzyme linked immunosorbent assay
GI: Gastrointestinal
H.pylori: Helicobacter pylori
MALT: Mucosal-associated lymphoid tissue
NUD: Non-ulcer dyspepsia
PCR: Polymerase chain reaction
RUT: Rapid urease test
UBT: Urea breath test
WGO: World Gastroenterology organization
WHO: World health organization
