# Supplementary material for: Helicobacter pylori infections in Ethiopia; prevalence and associated factors: a systematic review and meta-analysis
Source: BMC Gastroenterol. 2019 Jan 10;19:8. doi: 10.1186/s12876-018-0927-3 (PMC6327617; doi:10.1186/s12876-018-0927-3)
Supplement: Supplementary file 1 — Newcastle-Ottawa Scale for cross sectional studies. (DOC 34 kb) [file 12876_2018_927_MOESM1_ESM.doc]

**Newcastle-Ottawa Scale adapted for cross-sectional studies**

**Selection:** (Maximum 5 stars)

1) Representativeness of the sample:

a) Truly representative of the average in the target population. * (all subjects or random sampling)

b) Somewhat representative of the average in the target population. * (non-random sampling)

c) Selected group of users.

d) No description of the sampling strategy.

2) Sample size:

a) Justified and satisfactory. *

b) Not justified.

3) Non-respondents:

a) Comparability between respondents and non-respondents characteristics is established, and the response rate is satisfactory. *

b) The response rate is unsatisfactory, or the comparability between respondents and non-respondents is unsatisfactory.

c) No description of the response rate or the characteristics of the responders and the non-responders.

4) Ascertainment of the exposure (risk factor):

a) Validated measurement tool. **

b) Non-validated measurement tool, but the tool is available or described.*

c) No description of the measurement tool.

**Comparability:** (Maximum 2 stars)

1) The subjects in different outcome groups are comparable, based on the study design or analysis. Confounding factors are controlled.

a) The study controls for the most important factor (select one). *

b) The study control for any additional factor. *

**Outcome:** (Maximum 3 stars)

1) Assessment of the outcome:

a) Independent blind assessment. **

b) Record linkage. **

c) Self report. *

d) No description.

2) Statistical test:

a) The statistical test used to analyze the data is clearly described and appropriate, and the measurement of the association is presented, including confidence intervals and the probability level (p value). *

b) The statistical test is not appropriate, not described or incomplete.
